# Supplementary material for: Pacpaint: a histology-based deep learning model uncovers the extensive intratumor molecular heterogeneity of pancreatic adenocarcinoma
Source: Nat Commun. 2023 Jun 13;14:3459. doi: 10.1038/s41467-023-39026-y (PMC10264377; doi:10.1038/s41467-023-39026-y)
Supplement: Supplementary file 1 — Supplementary Information [file 41467_2023_39026_MOESM1_ESM.pdf]

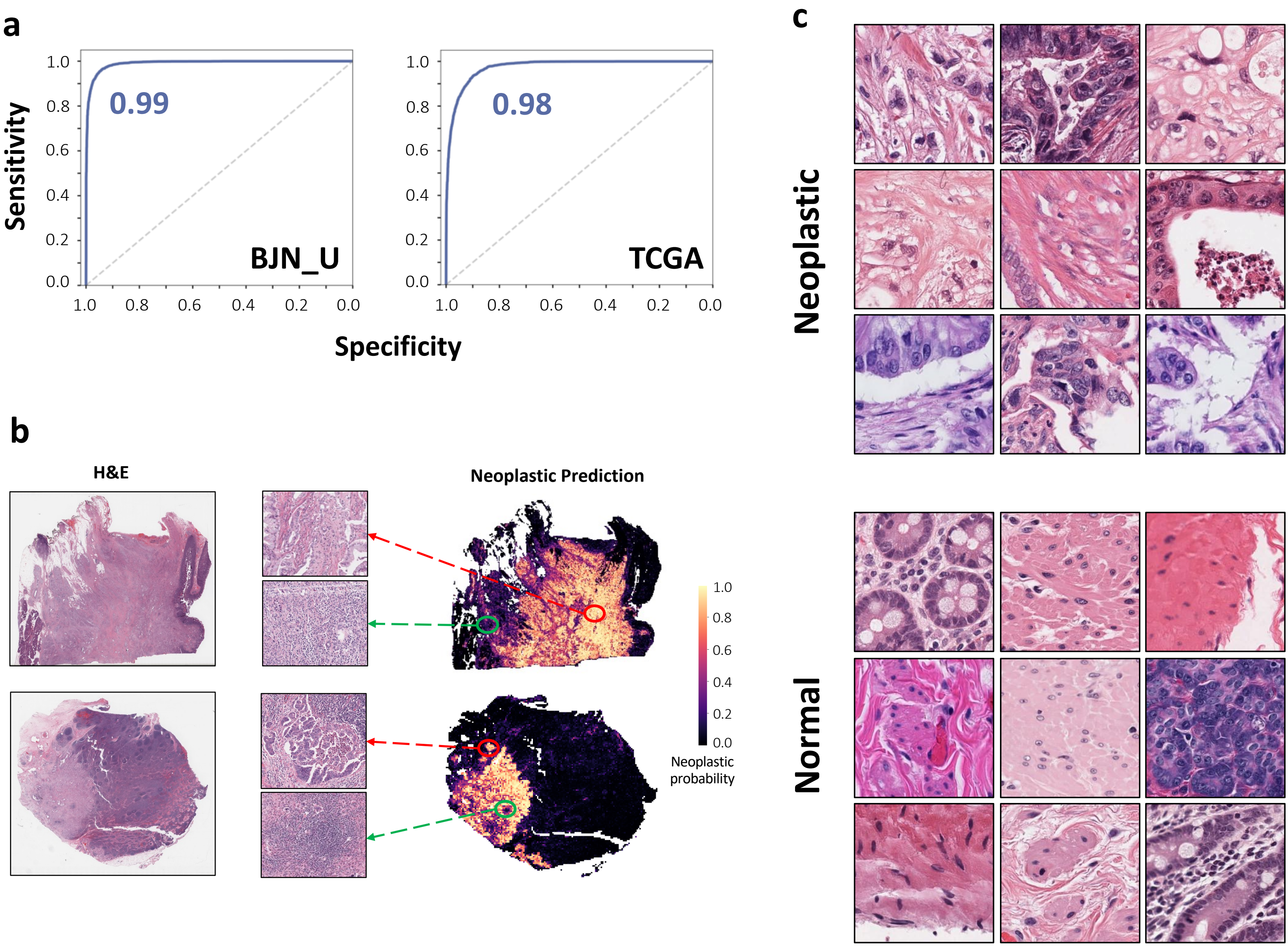

Supplementary Figure 1: Identification of neoplastic areas by PacpAInt-Neo. a) Performance of PACpAInt to identify neoplastic area in the BJN_U and TCGA validation cohorts, b) Example on 2 cases of neoplastic areas identification with H&E (left), PACpAInt-Neo segmentation (right) and zooms (center) of neoplastic (yellow) and non-neoplastic (green) areas, c) Representative tiles identified as neoplastic and non-neoplastic by PACpAInt-Neo in the TCGA validation cohort (112µm square). Source data are provided as a Source Data file.

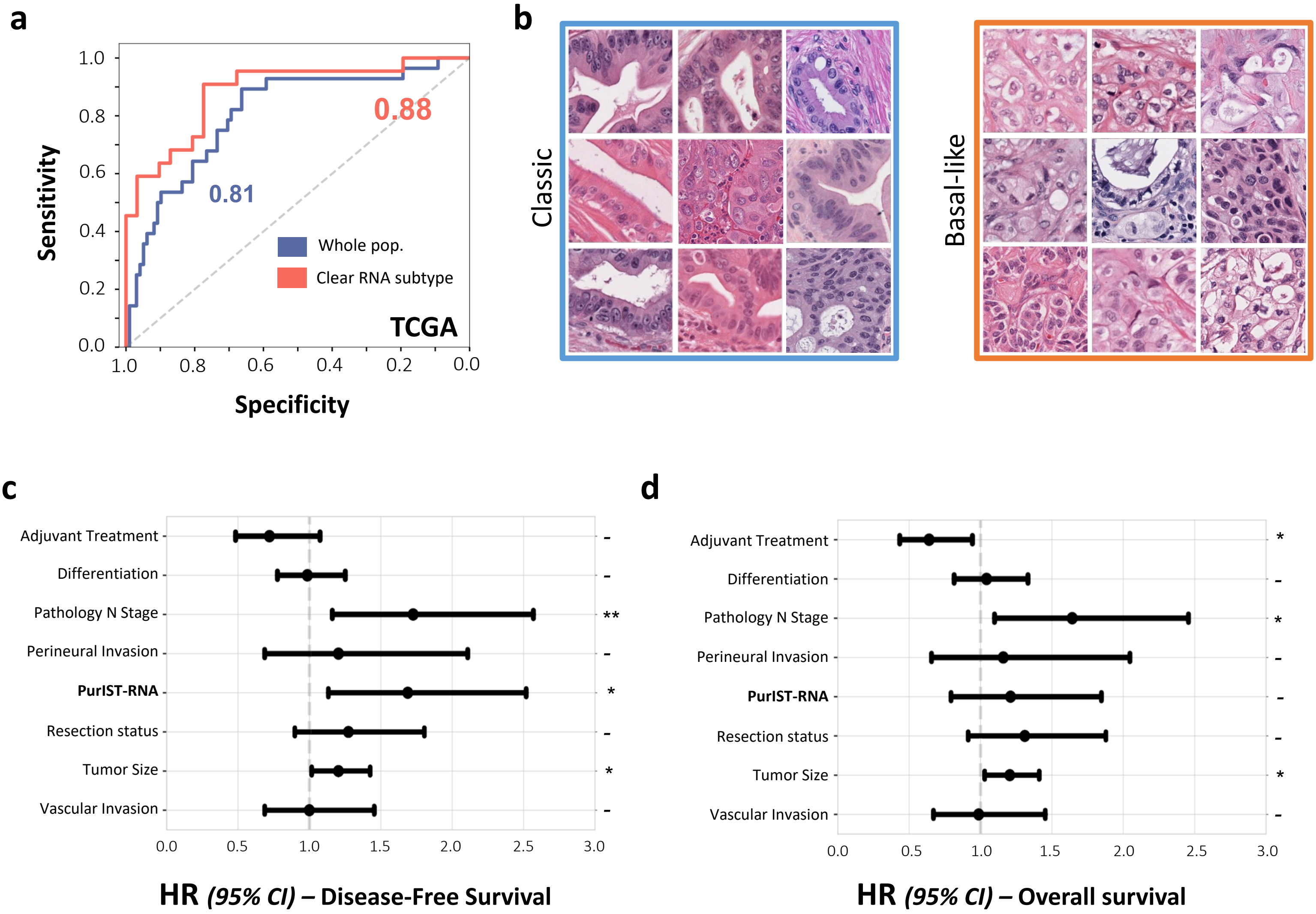

Supplementary Figure 2: Identification of the molecular subtypes at the whole slide level by PACpAInt-B/C in TCGA. a) Performance of PACpAInt-B/C to identify molecular subtypes at the whole slide level area in the TCGA validation cohort using the whole cohort or only cases with an unambiguous RNA subtype (clear subtype), b) Representative tiles identified as classical or basal-like by PACpAInt-B/C in the TCGA validation cohort, c/d) Multivariate analyses of clinical/pathological factors and RNA-defined molecular subtype (PurIST-RNA) on overall survival (top panel, n=243)) and disease free survival (bottom panel, n=248) in the BJN_U+M validation cohorts. p-values were computed using a two-sided Wald test. No adjustments for multiple comparisons were made. The circle represents the variable hazard ratio while whiskers represent the 95% confidence interval of that hazard ratio. p-values are for DFS Adjuvant Treatment, p=0.107657; Differentiation, p=0.903024; Pathology N Stage, p=0.007084; Perineural invasion, p=0.518108; PurIST-RNA, p=0.010171; Resection status, p=0.175429; Tumor Size, p=0.032051; Vascular Invasion, p=0.997119 and for OS Adjuvant Treatment, p=0.024104; Differentiation, p=0.741858; Pathology N Stage, p=0.015731; Perineural invasion, p=0.610226; PurIST-RNA, p=0.375653; Resection status, p=0.141991; Tumor Size, p=0.020583; Vascular Invasion, p=0.947557. ***: $p < 0.001$; ** : $p < 0.01$; * : $p < 0.05$; + : $p < 0.1$; − : $p > 0.1$. Source data are provided as a Source Data file.

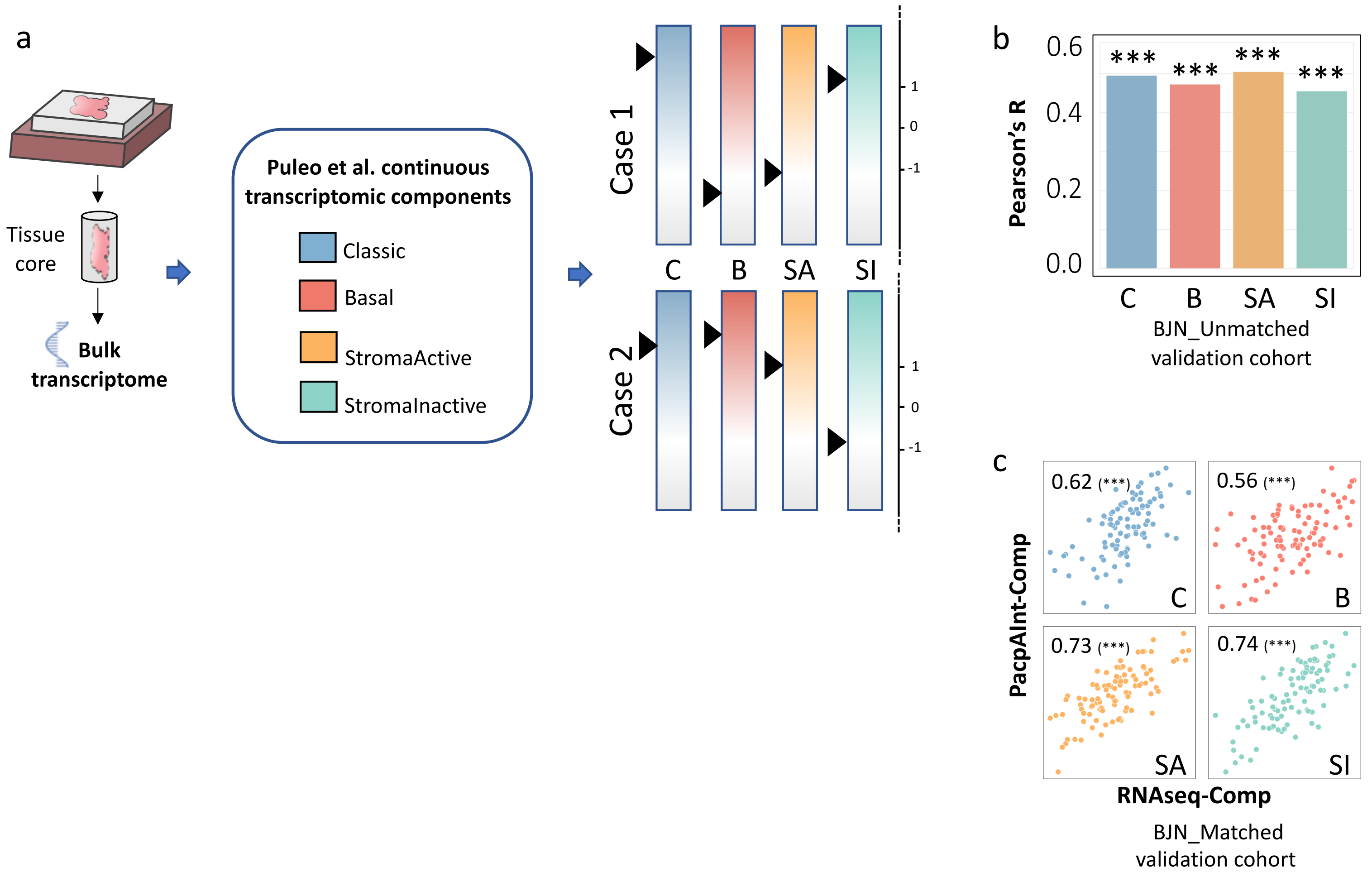

Supplementary Figure 3: Identification of the molecular subtypes of tumor cells and stroma at the whole slide level by PACpAInt-Comp. a) Schematic representation of the continuous RNA-based component approach to subtype PDAC tumor cell and stroma. Each tumor is scored for the four components (two on tumor cells: basal and classic and two on stroma (active and inactive) using RNA signatures. This approach acknowledge that a tumor can be homogeneous (top panel, high classical-low basal / high inactive-low active stroma) or heterogeneous (bottom panel high classical and high basal / high active stroma), b) Correlation at the slide level between the tumor and stromal components defined by RNAseq or PACpAInt-Comp on the BJN_U unmatched or BJN_M matched (c) validation cohorts. P-values were computed using two-sided t-tests. No adjustments for multiple comparisons were made. ***: P < 0.001. Source data are provided as a Source Data file.

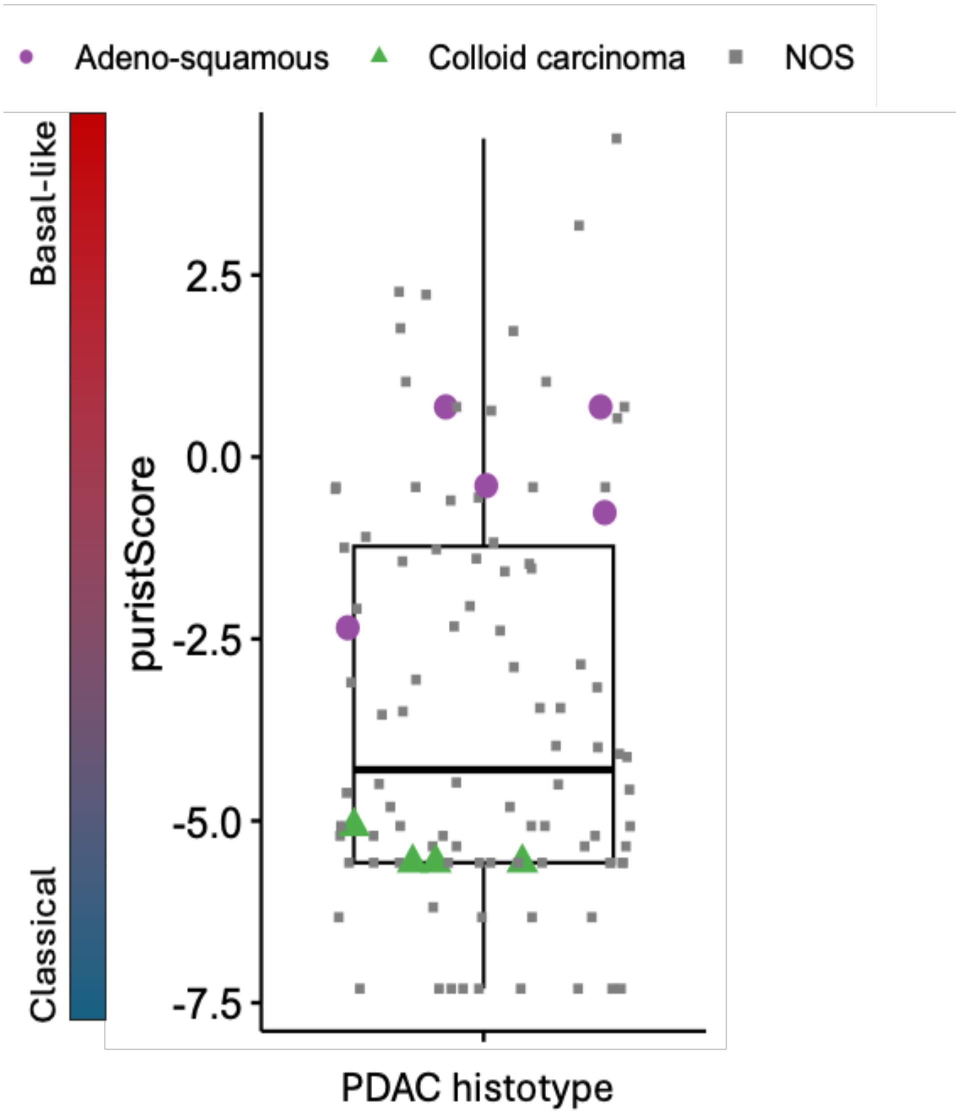

Supplementary Figure 4: Ranking according to the PurIST score of the 100 cases of the BJN_M with matched H&E and RNAseq. Colloid and adenosquamous carcinomas are highlighted by green triangles and purple dots respectively, demonstrating that these two morphological subtypes are well in line with the rest of the cases. Center corresponds to the median, lower and upper hingers to the first and third quartiles.

Cohort	Population	n	n Basal	n Classical	PACpAInt AUROC (p-value)
**BJN_U**	Whole population	148	32	116	0.861 [0.787-0.935] (p<1.0e-16)
	Well differentiated tumors	75	**7**	68	0.834 [0.706-0.962] (p=3.3e-7)
	Moderately differentiated tumors	47	12	35	0.771 [0.605-0.938] (p=0.00139)
	Poorly differentiated tumors	22	13	**9**	0.863 [0.708-1.000] (p=4.7e-5)
**BJN_M**	Whole population	97	22	75	0.833 [0.734-0.932] (p= 4.8e-11)
	Well differentiated tumors	38	**3**	34	0.706 [0.401-1.000] (p=0.18564)
	Moderately differentiated tumors	45	12	33	0.854 [0.721-0.986] (p=1.7e-7)
	Poorly differentiated tumors	14	6	**8**	0.896 [0.683-1.000] (p=0.00027)
**TCGA**	Whole population	126	28	98	0.806 [0.713-0.900] (p=1.5e-10)
	Well differentiated tumors	4	**1**	3	NA
	Moderately differentiated tumors	61	3	58	0.776 [0.597-0.955] (p=0.00249)
	Poorly differentiated tumors	60	24	**36**	0.797 [0.681-0.914] (p=5.4e-7)

Suppl Table 1: Performance of PACpAInt in the subgroups defined by differentiation in the different validation cohorts. P-values were computed using a Delong test. No adjustments for multiple comparisons were made.

Supplemental Table 2 : PACpAInt-B/C in BJN validation cohort

P-values were computed using a two-sided Wald test. No adjustments for multiple comparisons were made.

	Overall Survival		Disease-Free Survival	
**Covariate**	**HR (CI 95%)**	**p-value**	**HR (CI 95%)**	**p-value**
Differentiation	0.90 (0.70-1.16)	0.432849	0.96 (0.76-1.22)	0.757666
Vascular Invasion	0.93 (0.63-1.36)	0.696855	1.00 (0.69-1.44)	0.980046
Perineural Invasion	1.23 (0.70-2.17)	0.475994	1.26 (0.72-2.20)	0.423357
**Tumor Size**	1.29 (1.10-1.50)	0.001313	1.29 (1.08-1.53)	0.004102
**Pathology N Stage**	1.70 (1.14-2.53)	0.009221	1.74 (1.17-2.58)	0.005754
Resection Status	1.39 (0.97-2.00)	0.072018	1.26 (0.89-1.78)	0.196192
**Adjuvant Treatment**	0.63 (0.43-0.93)	0.020707	0.74 (0.50-1.11)	0.147249
**PACpAInt-B/C**	1.37 (1.16-1.62)	0.000218	1.27 (1.08-1.49)	0.003158

Supplemental table 3: PurIST-RNA in BJN validation cohort

P-values were computed using a two-sided Wald test. No adjustments for multiple comparisons were made.

	Overall Survival		Disease-Free Survival	
**Covariate**	**HR (CI 95%)**	**p-value**	**HR (CI 95%)**	**p-value**
Differentiation	1.04 (0.82-1.33)	0.741824	0.99 (0.78-1.25)	0.903024
Vascular Invasion	0.99 (0.67-1.45)	0.947547	1.00 (0.69-1.45)	0.997119
Perineural Invasion	1.16 (0.66-2.05)	0.610294	1.20 (0.69-2.11)	0.518107
**Tumor Size**	1.21 (1.03-1.41)	0.020576	1.20 (1.02-1.43)	0.032051
**Pathology N Stage**	1.64 (1.10-2.46)	0.015744	1.73 (1.16-2.57)	0.007084
Resection Status	1.31 (0.91-1.88)	0.141978	1.27 (0.90-1.81)	0.175429
**Adjuvant Treatment**	0.64 (0.43-0.94)	0.024102	0.72 (0.48-1.07)	0.107657
**PurIST-RNA**	1.21 (0.79-1.85)	0.375660	1.69 (1.13-2.52)	0.010171

Supplemental Table 4: PACpAInt-Cell type prognostic value

P-values were computed using a two-sided Wald test. No adjustments for multiple comparisons were made.

	Overall Survival		Disease-Free Survival	
**Covariate**	**HR (CI 95%)**	**p-value**	**HR (CI 95%)**	**p-value**
Differentiation	0.97 (0.81-1.16)	0.731474	1.02 (0.87-1.21)	0.779387
Vascular Invasion	1.08 (0.83-1.40)	0.558079	1.04 (0.81-1.34)	0.745010
Perineural Invasion	0.95 (0.68-1.32)	0.763570	1.06 (0.77-1.47)	0.721504
**Tumor Size**	1.12 (1.00-1.26)	0.055174	1.15 (1.02-1.30)	0.019459
**Pathology N Stage**	1.66 (1.22-2.25)	0.001374	1.67 (1.24-2.25)	0.000667
**Resection Status**	1.55 (1.18-2.05)	0.001951	1.52 (1.15-1.99)	0.002869
**Adjuvant Treatment**	0.66 (0.51-0.87)	0.002713	0.70 (0.53-0.91)	0.007784
**PurIST**	1.57 (1.19-2.07)	0.001470	1.68 (1.28-2.19)	0.000153
**PACpAInt-Cell type**	0.87 (0.77-0.98)	0.018986	0.86 (0.76-0.96)	0.008231

Supplemental table 5: Cox model with tile scores

Covariates	Overall Survival	Disease-Free Survival
Clinical variables	0.63	0.62
Clinical variables + PurIST	0.62	0.63
**Clinical variables + tile scores**	**0.67**	**0.65**

Supplemental table 6: PACpAInt-derived Basal proportion and its prognostic impact

P-values were computed using a two-sided Wald test. No adjustments for multiple comparisons were made.

	Overall Survival		Disease-Free Survival	
**Covariate**	**HR (CI 95%)**	**p-value**	**HR (CI 95%)**	**p-value**
Differentiation	0.94 (0.79-1.12)	0.494463	1.03 (0.87-1.22)	0.734589
Vascular Invasion	1.07 (0.83-1.39)	0.598309	1.05 (0.82-1.35)	0.709357
Perineural Invasion	0.94 (0.68-1.31)	0.734965	1.06 (0.77-1.47)	0.716473
**Tumor Size**	1.13 (1.01-1.26)	0.032572	1.16 (1.03-1.30)	0.012455
**Pathology N Stage**	1.65 (1.22-2.24)	0.001172	1.67 (1.25-2.24)	0.000521
**Resection Status**	1.36 (1.02-1.79)	0.033156	1.32 (1.00-1.75)	0.047713
**Adjuvant Treatment**	0.67 (0.52-0.88)	0.004106	0.72 (0.55-0.94)	0.015794
**Basal proportion**	1.36 (1.22-1.50)	0.000000	1.30 (1.17-1.45)	0.000001

Supplemental table 7: PACpAInt-derived subtypes and their prognostic impact

P-values were computed using a two-sided Wald test. No adjustments for multiple comparisons were made.

DFS	N	Event (n)	Median (months)	HR (Main class as ref)
**Main classical**	182	106	27.5 (22-38)	-
**Intermediary**	123	85	15.7 (12-19)	1.49 [1.12-1.99] p=0.006
**Hybrid**	42	31	12.3 (10-21)	1.75 [1.12-2.62] p=0.006
**Main basal**	80	67	8.4 (7-11)	2.40 [2.40-4.46] p=7.17e-14

OS	N	Event (n)	Median (months)	HR (Main class as ref)
**Main classical**	192	100	45.1 (39-63)	-
**Intermediary**	130	81	33.0 (25-36)	1.53 [1.14-2.05] p=0.005
**Hybrid**	45	30	23.4 (16-37)	1.99 [1.32-3.00] p=0.001
**Main basal**	844	65	13.6 (11-17)	3.74 [2.71-5.14] p=5.84e-16

Supplemental Table 8

TRIPOD Checklist: Prediction Model Development and Validation

Section/Topic	Item		Checklist Item	Page
**Title and abstract**				
Title	1	D;V	Identify the study as developing and/or validating a multivariable prediction model, the target population, and the outcome to be predicted.	1
Abstract	2	D;V	Provide a summary of objectives, study design, setting, participants, sample size, predictors, outcome, statistical analysis, results, and conclusions.	2
**Introduction**				
Background and objectives	3a	D;V	Explain the medical context (including whether diagnostic or prognostic) and rationale for developing or validating the multivariable prediction model, including references to existing models.	3, 4
	3b	D;V	Specify the objectives, including whether the study describes the development or validation of the model or both.	4
**Methods**				
Source of data	4a	D;V	Describe the study design or source of data (e.g., randomized trial, cohort, or registry data), separately for the development and validation data sets, if applicable.	14
	4b	D;V	Specify the key study dates, including start of accrual; end of accrual; and, if applicable, end of follow-up.	14
Participants	5a	D;V	Specify key elements of the study setting (e.g., primary care, secondary care, general population) including number and location of centres.	14
	5b	D;V	Describe eligibility criteria for participants.	14
	5c	D;V	Give details of treatments received, if relevant.	NA
Outcome	6a	D;V	Clearly define the outcome that is predicted by the prediction model, including how and when assessed.	17-19
	6b	D;V	Report any actions to blind assessment of the outcome to be predicted.	ND
Predictors	7a	D;V	Clearly define all predictors used in developing or validating the multivariable prediction model, including how and when they were measured.	14-16
	7b	D;V	Report any actions to blind assessment of predictors for the outcome and other predictors.	ND
Sample size	8	D;V	Explain how the study size was arrived at.	NA
Missing data	9	D;V	Describe how missing data were handled (e.g., complete-case analysis, single imputation, multiple imputation) with details of any imputation method.	NA
Statistical analysis methods	10a	D	Describe how predictors were handled in the analyses.	15, 16
	10b	D	Specify type of model, all model-building procedures (including any predictor selection), and method for internal validation.	17-19
	10c	V	For validation, describe how the predictions were calculated.	17,18
	10d	D;V	Specify all measures used to assess model performance and, if relevant, to compare multiple models.	19
	10e	V	Describe any model updating (e.g., recalibration) arising from the validation, if done.	NA
Risk groups	11	D;V	Provide details on how risk groups were created, if done.	19-20
Development vs. validation	12	V	For validation, identify any differences from the development data in setting, eligibility criteria, outcome, and predictors.	14
**Results**				
Participants	13a	D;V	Describe the flow of participants through the study, including the number of participants with and without the outcome and, if applicable, a summary of the follow-up time. A diagram may be helpful.	Fig. 1
	13b	D;V	Describe the characteristics of the participants (basic demographics, clinical features, available predictors), including the number of participants with missing data for predictors and outcome.	14,15
	13c	V	For validation, show a comparison with the development data of the distribution of important variables (demographics, predictors and outcome).	Suppl table 1
Model development	14a	D	Specify the number of participants and outcome events in each analysis.	Suppl table 1
	14b	D	If done, report the unadjusted association between each candidate predictor and outcome.	NA
Model specification	15a	D	Present the full prediction model to allow predictions for individuals (i.e., all regression coefficients, and model intercept or baseline survival at a given time point).	NA
	15b	D	Explain how to the use the prediction model.	16
Model performance	16	D;V	Report performance measures (with CIs) for the prediction model.	6, 8
Model-updating	17	V	If done, report the results from any model updating (i.e., model specification, model performance).	NA
**Discussion**				
Limitations	18	D;V	Discuss any limitations of the study (such as nonrepresentative sample, few events per predictor, missing data).	11-13
Interpretation	19a	V	For validation, discuss the results with reference to performance in the development data, and any other validation data.	11-13
	19b	D;V	Give an overall interpretation of the results, considering objectives, limitations, results from similar studies, and other relevant evidence.	11-13
Implications	20	D;V	Discuss the potential clinical use of the model and implications for future research.	11-13
**Other information**				
Supplementary information	21	D;V	Provide information about the availability of supplementary resources, such as study protocol, Web calculator, and data sets.	16,17
Funding	22	D;V	Give the source of funding and the role of the funders for the present study.	NA

		Cohort		P-value
**Variable**	**Category**	**Discovery (DISC)**	**Validation (BJN_M & BJN_U)**	
**Number of patients**	-	202	245	-
**Number of centers**	-	3	1	-
**PurIST**	Classical	149 (73%)	191 (77%)	0.36
	Basal	53 (26%)	54 (22%)	
**Age at Surgery IQR**	-	63 [57, 72]	63 [56, 71]	0.44
**Gender**	Female	81 (40%)	130 (53%)	0.008
	Male	121 (59%)	115 (46%)	
**Differentiation**	Good	81 (40%)	111 (45%)	0.49
	Moderate	85 (42%)	93 (37%)	
	Poor	26 (12%)	37 (15%)	
**Resection Status**	R0	163 (80%)	177 (72%)	0.013
	R1	34 (16%)	68 (27%)	
**Tumor Size (mm) IQR**	-	30 [24, 40]	30 [23, 40]	0.97
**Lymph Node stage**	N0	49 (24%)	64 (26%)	0.73
	N+	153 (75%)	181 (73%)	
**Overall Survival**	Median Survival	30 months	35 months	0.12
**Disease-Free Survival**	Median Survival	15 months	17 months	0.20

Supplemental Table 9: Comparison of pathological and clinical data of the test and validation cohorts. P-values were computed for categorical variables using a chi-square test of independence. P-values were computed for continuous variables using a two-sided t-test. No adjustments for multiple comparisons were made.
