# Supplementary material for: Pacpaint: a histology-based deep learning model uncovers the extensive intratumor molecular heterogeneity of pancreatic adenocarcinoma
Source: Nat Commun. 2023 Jun 13;14:3459. doi: 10.1038/s41467-023-39026-y (PMC10264377; doi:10.1038/s41467-023-39026-y)
Supplement: Supplementary file 3 — Reporting Summary [file 41467_2023_39026_MOESM3_ESM.pdf]

nature portfolio

Corresponding author(s): Jerome CROS

Last updated by author(s): 30_03_2023

Reporting Summary

Nature Portfolio wishes to improve the reproducibility of the work that we publish. This form provides structure for consistency and transparency in reporting. For further information on Nature Portfolio policies, see our Editorial Policies and the Editorial Policy Checklist.

Statistics

For all statistical analyses, confirm that the following items are present in the figure legend, table legend, main text, or Methods section.

n/a	Confirmed	
☐	☒	The exact sample size (*n*) for each experimental group/condition, given as a discrete number and unit of measurement
☐	☒	A statement on whether measurements were taken from distinct samples or whether the same sample was measured repeatedly
☐	☒	The statistical test(s) used AND whether they are one- or two-sided<br>*Only common tests should be described solely by name; describe more complex techniques in the Methods section.*
☐	☒	A description of all covariates tested
☒	☐	A description of any assumptions or corrections, such as tests of normality and adjustment for multiple comparisons
☐	☒	A full description of the statistical parameters including central tendency (e.g. means) or other basic estimates (e.g. regression coefficient) AND variation (e.g. standard deviation) or associated estimates of uncertainty (e.g. confidence intervals)
☐	☒	For null hypothesis testing, the test statistic (e.g. *F*, *t*, *r*) with confidence intervals, effect sizes, degrees of freedom and *P* value noted<br>*Give P values as exact values whenever suitable.*
☒	☐	For Bayesian analysis, information on the choice of priors and Markov chain Monte Carlo settings
☐	☒	For hierarchical and complex designs, identification of the appropriate level for tests and full reporting of outcomes
☐	☒	Estimates of effect sizes (e.g. Cohen's *d*, Pearson's *r*), indicating how they were calculated

Our web collection on statistics for biologists contains articles on many of the points above.

Software and code

Policy information about availability of computer code

Data collection	Transcriptome data was directly downloaded from public repositories. RNAseq were processed using STAR 2.7.5a and featureCount on Ensembl genome and transcriptome annotation.
Data analysis	PACpAInt, a commercial software owned by Owkin, was used to classify and quantify pancreatic adenocarcinoma phenotypes. The code can be accessed here : https://github.com/owkin/pacpaint

For manuscripts utilizing custom algorithms or software that are central to the research but not yet described in published literature, software must be made available to editors and reviewers. We strongly encourage code deposition in a community repository (e.g. GitHub). See the Nature Portfolio guidelines for submitting code & software for further information.

Data

Policy information about availability of data

All manuscripts must include a data availability statement. This statement should provide the following information, where applicable:

- Accession codes, unique identifiers, or web links for publicly available datasets
- A description of any restrictions on data availability
- For clinical datasets or third party data, please ensure that the statement adheres to our policy

The datasets generated during and/or analysed during the current study are available in the TCGA repository (TCGA_PAAD, https://portal.gdc.cancer.gov) and in the Geo dataset repository under the accession number GSE85916 for the microarray data. RNAseq data are available here : array express (E-MTAB-13007).

Human research participants

Policy information about studies involving human research participants and Sex and Gender in Research.

Reporting on sex and gender	We only used the biological sex and not the gender in this study and results were presented as such.
Population characteristics	Inclusion criteria for all cohorts were as follows: unequivocal diagnosis of the most common histological variants of pancreatic adenocarcinoma (i.e ductal, adenosquamous and colloid carcinomas), available histological slides of formalin-fixed, paraffin-embedded material, available follow-up and molecular information, absence of metastasis at diagnosis.
Recruitment	Patients were recruited restrospectively from the pathology databases based on the inclusion criteria mentioned above.
Ethics oversight	This study (ref 2020-013) was reviewed and approved by the "Comite d'Evaluation de l'Ethique des projets de Recherche Biomedicale (CEERB) Paris Nord" (Institutional Review Board -IRB 00006477- of HUPNVS, Paris 7 University, AP-HP). Non-deceased patients were informed in writing of the study. According to the French Jardé Law for non-interventional studies they had a two-month period to express in writing their opposition to the study. No patient declined the study. Patients were not compensated for their participation in the study.

Note that full information on the approval of the study protocol must also be provided in the manuscript.

Field-specific reporting

Please select the one below that is the best fit for your research. If you are not sure, read the appropriate sections before making your selection.

☒ Life sciences ☐ Behavioural & social sciences ☐ Ecological, evolutionary & environmental sciences

For a reference copy of the document with all sections, see nature.com/documents/nr-reporting-summary-flat.pdf

Life sciences study design

All studies must disclose on these points even when the disclosure is negative.

Sample size	For all cohorts, sample sizes were determined based on the maximum number of samples available which respect the inclusion criteria detailed below.
Data exclusions	Inclusion criteria for all cohorts were as follows: unequivocal diagnosis of Pancreatic Adenocarcinoma (PAC) without neoadjuvant treatment, available histological slides of formalin-fixed, paraffin-embedded material, available follow-up and molecular information, absence of metastasis at diagnosis. Data which does not respect these criteria were excluded from the study.
Replication	The results have been generated via a python script to guarantee that they can be reproduced easily.
Randomization	Patient splits for cross-validation was performed at random. For PurIST prediction, cross-validation splits were furthermore stratified on positive class.
Blinding	Pathologists were independently assigned regions of interest to review and were not able to communicate on their results to each other so that there is no bias in each pathologist review.

Reporting for specific materials, systems and methods

We require information from authors about some types of materials, experimental systems and methods used in many studies. Here, indicate whether each material, system or method listed is relevant to your study. If you are not sure if a list item applies to your research, read the appropriate section before selecting a response.

Materials & experimental systems

n/a	Involved in the study	
☐	☒	Antibodies
☒	☐	Eukaryotic cell lines
☒	☐	Palaeontology and archaeology
☒	☐	Animals and other organisms
☒	☐	Clinical data
☒	☐	Dual use research of concern

Methods

n/a	Involved in the study	
☒	☐	ChIP-seq
☒	☐	Flow cytometry
☒	☐	MRI-based neuroimaging

Antibodies

Antibodies used

The following antibodies were used (GATA6 (Cell Signaling, clone D61E4, Rabbit, at 1/200, REF5158S), Claudin18 (Sigma, Polyclonal ref HPA018446, Rabbit, at 1/50, REF HPA01846), KRT17 (BioSB, clone BSB-33, mouse, at 1/800, REF BSB2729), PanCK+ (Zytomed, clone coktail AE1/AE3/5D3, mouse, at 1/300, REF MSK098-05).

Validation

All the antibodies were tested in the diagnostic pathology department of an expert center in pancreatic diseases (Beaujon hospital) and verified by J. Cros, a certified pancreatic pathologist. For each antibody, the proper staining was verified (proper stained cell and proper localisation of the staining (nucleus, cytoplasm etc...)). All staining were performed on a routine automate (Ventana Benchmark ultra). The GATA6 antibody is a monoclonal antibody from a vendor with a good reputation and is the most widely used (84 citations). The Claudin 18 antibody is a rabbit polyclonal but is a Prestige Antibodies® Powered by Atlas Antibodies (tested in multiples tissues againts RNAseq data to confirm its specificity. Every Prestige Antibody is tested in the following ways:
IHC tissue array of 44 normal human tissues and 20 of the most common cancer type tissues and protein array of 364 human recombinant protein fragments. The KRT17 and PanCK+ antibodies have the CE/IVD label (validated for Vitro Diagnostic use) ensuring their stability and accuracy.
